# *Bacillus anthracis* Secretes Proteins That Mediate Heme Acquisition from Hemoglobin

**DOI:** 10.1371/journal.ppat.1000132

**Published:** 2008-08-22

**Authors:** Anthony W. Maresso, Gabriella Garufi, Olaf Schneewind

**Affiliations:** Department of Microbiology, University of Chicago, Chicago, Illinois, United States of America; The University of Texas-Houston Medical School, United States of America

## Abstract

Acquisition of iron is necessary for the replication of nearly all bacterial pathogens; however, iron of vertebrate hosts is mostly sequestered by heme and bound to hemoglobin within red blood cells. In *Bacillus anthracis*, the spore-forming agent of anthrax, the mechanisms of iron scavenging from hemoglobin are unknown. We report here that *B. anthracis* secretes IsdX1 and IsdX2, two NEAT domain proteins, to remove heme from hemoglobin, thereby retrieving iron for bacterial growth. Unlike other Gram-positive bacteria, which rely on cell wall anchored Isd proteins for heme scavenging, *B. anthracis* seems to have also evolved NEAT domain proteins in the extracellular milieu and in the bacterial envelope to provide for the passage of heme.

## Author Summary

Iron is an essential nutrient used by almost all organisms. Bacterial pathogens must acquire iron in order to grow inside mammalian hosts. The host, however, limits the availability of free iron, thereby providing an effective defense strategy against infection. In response, bacteria have evolved clever ways to subvert host sequestration of iron. In this work, we report that the causative agent of anthrax disease, *Bacillus anthracis*, produces two proteins (IsdX1 and IsdX2), which act to acquire iron complexed to heme, a co-factor of host hemoproteins such as hemoglobin. This activity is dependent on a conserved protein domain found in many Gram-positive bacterial pathogens and is necessary for growth of *B. anthracis* in low-iron environments. Our results yield a greater understanding of the mechanisms used by bacterial pathogens to subvert host defenses and provide an avenue for the development of antiinfectives that aim to block these strategies.

## Introduction

Vegetative forms of *Bacillus anthracis* replicate in vertebrate tissues and form spores once their host has succumbed to anthrax infection [1]. Spore contamination of food sources for vertebrates ensures pathogen dissemination to new hosts and reiterative replication cycles [2]. A hallmark of anthrax is its low infectious dose (25–50 spores can kill an animal) and explosive replication of vegetative forms that accumulate to 10^10^ colony forming units (CFU) per gram of host tissue [3]. Spores are taken up by phagocytes and germinate in the phagosome [4],[5]. Upon phagosome lysis, vegetative forms first multiply in the cytoplasm, however, once released into body fluids, bacilli resist phagocytosis and replicate in extracellular spaces [6].

Several key features enable the invasion and replication strategies of *B. anthracis*. First, spores are metabolically inert and survive in the environment for long periods of time until taken up by a new host [7]. To escape phagocyte killing, bacilli secrete lethal toxin and edema toxin that subvert the host immune system and implement host killing [8]. Elaboration of the dense poly-D-glutamic acid (PDGA) capsule endows vegetative forms with the characteristic trait of resisting phagocytosis [9]. PDGA is attached to peptidoglycan [10], which functions as an exoskeletal scaffold for immobilization of proteins, carbohydrates and the S-layer, a two-dimensional crystalline protein array that encases vegetative forms [11],[12].

**Figure 1:**
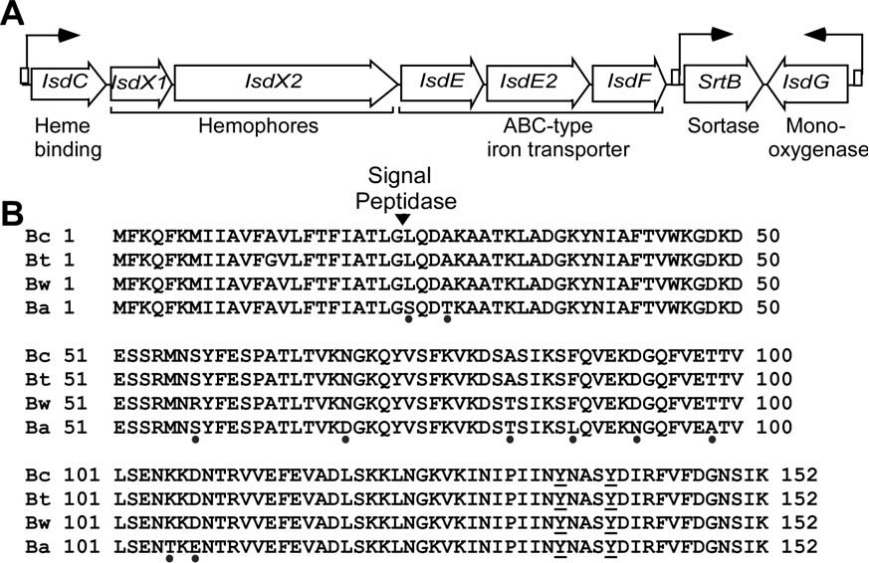
*Bacillus anthracis isdX1* and *isdX2*. (A) The *B. anthracis isd* locus contains eight open reading frames, including genes for sortase B (*srtB*), IsdC (a NEAT domain protein and sortase B substrate), IsdE1-IsdE2-IsdF (ABC membrane transporter), IsdG (heme mono-oxygenase), and two NEAT domain proteins of unknown function (IsdX1 and IsdX2). (B) Alignment of amino acid sequences of *B. anthracis* IsdX1 (Ba - BAS4443) with homologs from *B. cereus* (Bc - BC4548), *B. thuringiensis* (Bt - RBTH03454), and *B. weihenstephanensis* (Bw - KBAB44137). Arrow indicates the predicted signal peptide cleavage site. Black dots refer to amino acids that are not absolutely conserved. Amino acids 27–152 of IsdX1 represent a NEAT (near iron transporter) domain. Tyrosine residues 136 and 140, which are conserved in many NEAT proteins, are underlined.

Heme scavenging has been studied in *Staphylococcus aureus*, a Gram-positive pathogen phylogenetically related to *B. anthracis*, albeit that the envelope structure of staphylococci is comprised entirely of cell wall peptidoglycan with associated protein, teichoic acid and carbohydrate polymers [13],[14]. Staphylococci elaborate neither PDGA capsule nor S-layers and their ability to retrieve heme from hemoglobin/haptoglobin relies on Isd proteins that are anchored to cell wall peptidoglycan [15],[16]. The *S. aureus isd* locus (*isdA-isdB-isdCDEF srtB isdG*) is comprised of genes that encode cell wall anchored surface proteins (IsdA, IsdB, IsdC), membrane protein (IsdD), ABC transporter for import of heme (IsdEF) as well as heme mono-oxygenase (IsdG) [17],[18]. The NEAT domain (near iron transporter) of staphylococcal envelope proteins (IsdA, IsdB, IsdC) enables scavenging of heme and passage of the iron containing compound across the cell wall envelope [15],[16],[19],[20],[21]. Heme passage relies further on sortase A-mediated deposition of IsdA and IsdB at the bacterial surface as well as sortase B-mediated immobilization of IsdC within the cell wall envelope [15],[22],[23]. The *B. anthracis isd* locus (*isdC isdX1 isdX2 isdE1 isdE2 isdF-srtB-isdG*) is comprised of eight open-reading frames with three putative transcriptional units, each flanked by a Fur-box consensus sequence (Fig. 1A) [24],[25]. The smallest gene, *isdX1*, harbors a NEAT domain and is conserved in all members of the *Bacillus cereus* group but absent from staphylococci, listeria and clostridia (Fig. 1B). The largest gene, *isdX2*, is also conserved and contains five NEAT domains.

Here we report the first identification of a secreted heme-scavenging protein, IsdX1, from Gram-positive bacteria. Further, we demonstrate that IsdX1 and IsdX2 acquire heme directly from hemoglobin and that this activity enables bacilli to scavenge iron from host hemoglobin under iron-limiting conditions. These findings indicate that unlike staphylococci, which rely on cell wall anchored Isd proteins for heme scavenging, *B. anthracis* seems to have also evolved NEAT domain proteins in the extracellular milieu and in the bacterial envelope to provide for the passage of heme.

## Results

### *Bacillus anthracis* secretes IsdX1

**Figure 2:**
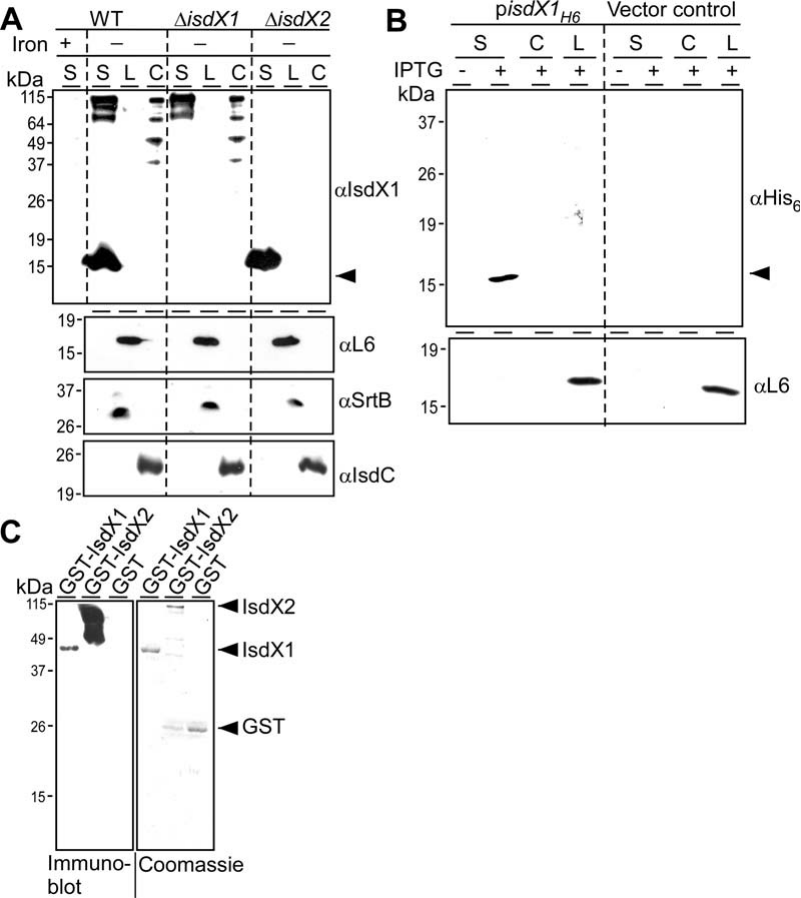
*B. anthracis* secretes IsdX1. (A and B) *B. anthracis* strains were grown in iron-replete or iron-depleted medium for 12 hours, followed by fractionation of cultures into secreted (S), cell wall (C), and lysate (L) fractions. Samples were analyzed by immunoblot with antibodies specific for IsdX1, L6 (ribosomal protein), SrtB (sortase B membrane protein), and IsdC (cell wall anchored protein). (C) Serum of guinea pigs that recovered from anthrax infections was used for immunoblot with purified GST-IsdX1, GST-IsdX2 or GST (*left panel*). The arrows denote SDS-PAGE mobilities of proteins (Coomassie, *right panel*) used for the immunoblot analysis. The migratory positions of molecular weight markers are indicated (kDa).

The presence of cleavable N-terminal signal peptides and the absence of membrane or cell wall anchoring signals suggested that IsdX1 and IsdX2 may be secreted. To test this, *B. anthracis* was grown in the presence or absence of iron and bacterial cultures were fractionated to separate proteins secreted into the medium (S) from those targeted to the cell wall envelope (C) or located in membrane and cytoplasm lysate (L) (Fig. 2A). When analyzed by immunoblotting with rabbit antiserum raised against purified recombinant IsdX1, 15 kDa and 100 kDa (including some degradation products) immunoreactive species were detected under iron-limiting conditions. Wild-type bacilli secreted both the 15 and 100 kDa proteins, which represent IsdX1 (predicted molecular mass 14,579) and IsdX2 (predicted molecular mass 99,610), as Δ*isdX1* and Δ*isdX2* mutant strains failed to express the former or the latter species, respectively (Fig. 2A). Cross-reactivity of IsdX1 was not observed for other NEAT domain proteins, suggesting that IsdX1 and IsdX2 may share unique structural and functional properties (data not shown). A portion of IsdX2, but not of IsdX1, was found in the cell wall fraction [24% (±9) of the total], suggesting that IsdX2 may be partially associated with the envelope of bacilli. As a control, immunoblotting with antibodies against cell wall anchored (IsdC), membrane (SrtB) and cytoplasmic (L6) proteins was used to ensure proper fractionation of *B. anthracis* cultures (Fig. 2A). The amount of IsdX1 or IsdX2 secretion was similar when bacilli were grown at 30°C or 37°C (Fig. S1). Taken together, these data indicate that IsdX1 and IsdX2 are synthesized and secreted when bacilli are exposed to iron-limiting conditions, as occurs during infection of vertebrate hosts. *isdX1* with a C-terminal hexahistidyl tag was cloned under control of the IPTG inducible P*_spac_* promoter in pLM5 and recombinant plasmid was transformed into bacilli. Affinity blotting of fractionated cultures revealed that bacilli harboring p*isdX1-H6*, but not bacteria harboring pLM5 vector control, secreted IsdX1_H6_ into the extracellular milieu (Fig. 2B).

### Host immune responses to *B. anthracis* IsdX1 and IsdX2

To test whether *B. anthracis* synthesize IsdX1 and IsdX2 during infection, we analyzed the serum of guinea pigs that had survived anthrax infections. Following subcutaneous infection with spores of *B. anthracis* strain Ames, guinea pigs suffer lethal anthrax infections over seven days, even when animals are inoculated with low doses of spores [26]. To ensure survival of guinea pigs, animals were treated with ciprofloxacin five days following infection, at a time when spores had germinated and vegetative bacilli replicated throughout host tissues. Two weeks following infection, animals were bled and serum samples examined for the presence of antibodies against purified recombinant GST-IsdX1, GST-IsdX2 or a GST control. Immune sera from infected animals reacted with GST-IsdX1, and GST-IsdX2, but not with the GST control (Fig. 2C). These data suggest that *B. anthracis* secretes IsdX1 and IsdX2 during infection when vegetative forms encounter iron-restrictive conditions, thereby stimulating specific host immune responses against these proteins.

### IsdX1 binds heme

**Figure 3:**
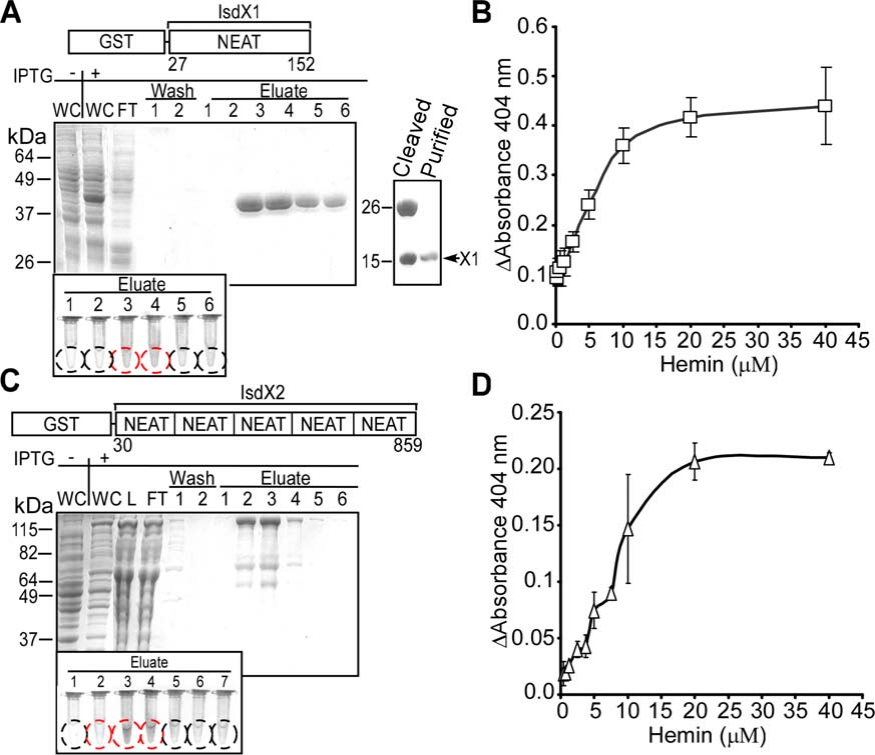
IsdX1 binds heme. (A and C) Codons 27–152 of *isdX1* and 30–859 of *isdX2* were cloned into pGEX-2TK and the hybrid GST-fusions purified by affinity chromatography. Whole cells (WC), flow through (FT), wash and eluate fractions were analyzed on Coomassie-stained SDS-PAGE. The arrow in A identifies IsdX1, where GST had been removed with thrombin. The insets display tubes with eluate fractions (circled) containing red-brown pigment indicative of iron-porphyrin binding (red circles). (B and D) IsdX1 (20 µM) or IsdX2 (1 µM) were incubated with hemin and absorbance at 404 nm measured. Mean and standard deviation of three independent experiments were recorded.

Several NEAT-domain containing proteins have been shown to bind heme, including *B. anthracis* IsdC (B-IsdC) [15],[20],[21],[25]. To determine whether IsdX1 and IsdX2 display a similar property, both genes were cloned as translational fusions to the 3′ end of glutathione-S-transferase (*gst*) and GST-IsdX1/-IsdX2 purified from *E. coli* lysate by affinity chromatography (Fig. 3AC). Both GST-IsdX1 and GST-IsdX2 eluted with red-brown color, indicative of an association with endogenous iron-porphyrin from *E. coli* (Fig. 3AC *insets*) [25]. We estimate that about 10% of purified GST-IsdX1/-IsdX2 was bound to heme [27]. GST-IsdX1 was dialyzed to remove heme, cleaved with thrombin and IsdX1 purified (Fig. 3A). Binding of added heme to IsdX1, as analyzed by spectrophotometry (Soret absorbance at 404 nm) [28], was dose-dependent and quantifiable (*K_d_* 5.40±0.85×10^−6^ M) (Fig. 3B). Heme binding was only marginally increased by an increase in temperature (Fig. S2). IsdX2 also bound heme in a dose-dependent manner and did so more efficiently than IsdX1 (Fig. 3D). The heme binding curve of IsdX2 yielded multiple inflection points, suggesting IsdX2 contains multiple binding sites for heme, presumably provided by its five NEAT domains. The complexity of the associations between IsdX2 and heme did not allow us to calculate a dissociation constant (Fig. 3D). Together these findings indicate that IsdX1 and IsdX2 bind heme and may be involved in iron scavenging during anthrax infections.

### IsdX1 removes heme from hemoglobin

**Figure 4:**
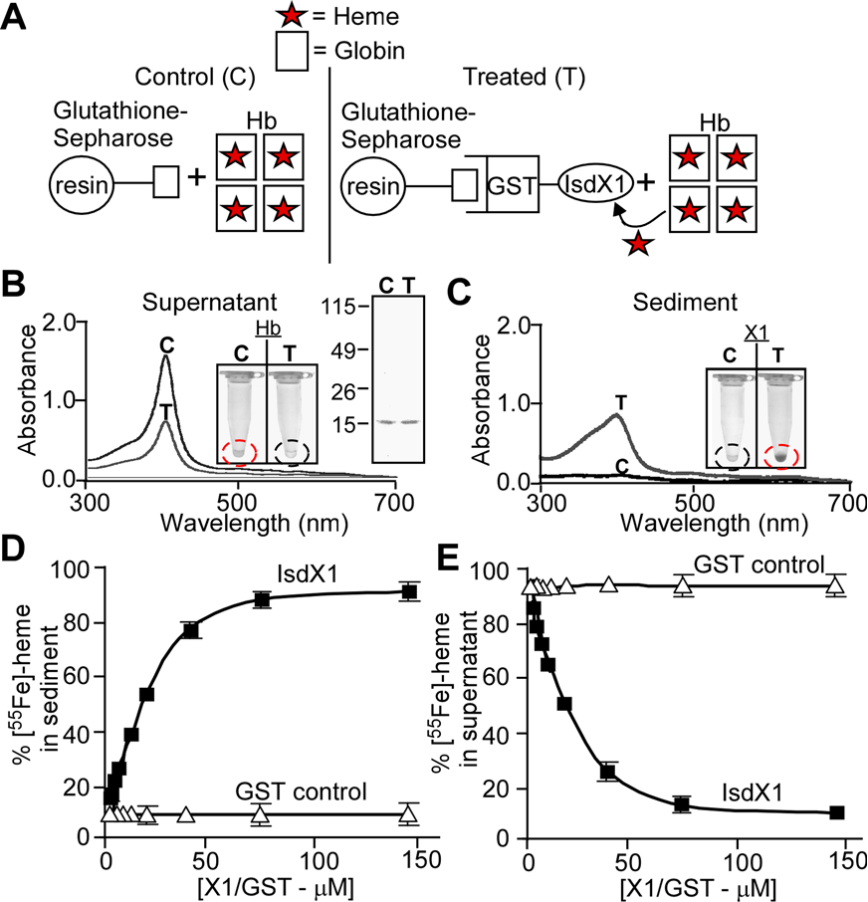
IsdX1 removes heme from hemoglobin. (A) Illustration of heme-transfer assay. Red stars indicate heme. (B, C) Glutathione-sepharose charged with GST-IsdX1 or GST (60 µM) was incubated with equimolar amounts of hemoglobin for 30 min, followed by centrifugation. Supernatant (B) and sediment (C) were assayed for absorbance at 404 nm. Insets display heme pigment in tubes with supernatant or sediment for GST-IsdX1 treated (red circles) or GST control (black circles). (D, E) Heme transfer was measured by adding increasing amounts of GST-IsdX1 or GST control (loaded on glutathione sepharose) to [^55^Fe-heme]hemoglobin and radioactivity in sediment and supernatant samples recorded. Mean and standard deviation of three independent experiments are indicated.

Hemoglobin (Hb) is the most abundant hemoprotein of mammals and several bacterial pathogens target this molecule to obtain iron during infection [29],[30]. To examine whether hemoglobin serves as a source of heme for the presumed iron-scavenging activity of IsdX1, we developed a simple experimental protocol. Glutathione-sepharose loaded with GST-IsdX1 was incubated with hemoglobin. The resin was then sedimented by centrifugation, separated from supernatant containing hemoglobin, washed and GST-IsdX1 eluted (Fig. 4A). As a control (C), hemoglobin was incubated with glutathione-sepharose that had been charged with GST and compared with GST-IsdX1 treated samples (T) (Fig. 4BC). Following incubation with GST-IsdX1, the heme-specific absorbance of hemoglobin at 404 nm was diminished, indicating that GST-IsdX1 had removed heme from hemoglobin (Fig. 4B). GST-IsdX1 mediated removal of heme could also be observed by inspection of hemoglobin: the red-brown color of hemoglobin is cleared in GST-IsdX1 treated, but not in GST control samples (inset, Fig. 4B). When analyzed by spectrophotometry, GST-IsdX1 displayed an increase in absorbance at 404 nm following its incubation with hemoglobin (Fig. 4C). Inspection of glutathione sepharose sediment revealed red-brown pigmented GST-IsdX1, whereas GST control samples remained clear (inset, Fig. 4C). When analyzed by spectrophotometry, GST-IsdX1 displayed an increase in absorbance at 404 nm following its incubation with hemoglobin (Fig. 4C). The abundance of hemoglobin in the supernatant samples was unchanged in the treated versus control reactions, indicating that the observed color and spectral changes were caused by heme transfer to IsdX1 (Coomassie stained SDS-PAGE, Fig. 4B).

We sought to develop a second measure for GST-IsdX1 removal of heme from hemoprotein. Apo-hemoglobin (hemoglobin lacking heme) was loaded with [^55^Fe]heme and radiolabeled hemoglobin was purified. [^55^Fe]hemoglobin was incubated with GST or GST-IsdX1 bound to glutathione-sepharose. As before, glutathione sepharose was sedimented by centrifugation and transfer of [^55^Fe]heme from hemoglobin was measured by scintillation counting as an increase in [^55^Fe]ionization (Fig. 4D). Addition of increasing amounts of GST-IsdX1, but not of GST, to [^55^Fe]hemoglobin led to increased [^55^Fe]ionization in sediment samples, until eventually all [^55^Fe]heme had been removed from hemoglobin (Fig. 4E) and transferred to GST-IsdX1 (Fig. 4D).

### Comparison of *B. anthracis* IsdX1 and *S. marcescens* HasA

**Figure 5 ppat-1000132-g005:**
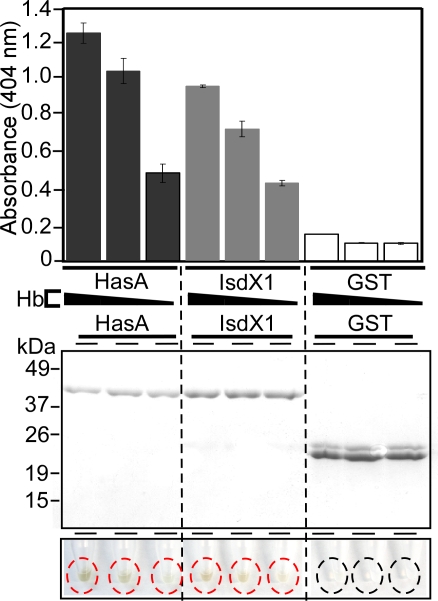
Heme transfer from hemoglobin to IsdX1 or HasA. HasA is a known hemophore in the Gram-negative pathogen *Serratia marcescens*. Glutathione-sepharose charged with GST-HasA, GST-IsdX1 or GST (200 µM) was incubated with increasing amounts of hemoglobin (50, 200, or 800 µM) for 30 min, followed by centrifugation. Sediment and supernatant samples were assayed for absorbance at 404 nm. Middle panel - Coomassie stained SDS-PAGE reveals GST proteins eluted from glutathione-sepharose. Lower panel - displays heme pigment in tubes with sediment derived from GST-HasA (red circles), GST-IsdX1 (red circles) or GST (black circles) treated hemoglobin samples. Mean and standard deviation of three independent experiments are recorded.

*Serratia marcescens* HasA represents the best established paradigm of bacterial hemophores [31]. Following its secretion via the *Serratia* type I pathway, 19 kDa HasA binds heme (*K_a_* 5×10^10^ M^−1^) [32],[33]. Due to its high affinity, HasA retrieves heme from hemoglobin and, in turn, transfers heme to the HasR outer membrane receptor for heme transport across the bacterial envelope and into the cytosol [34]. To validate our heme-transfer assay as a method to measure heme transfer between proteins, we compared the ability of IsdX1 to acquire heme from hemoglobin with that of HasA. We purified GST-HasA from lysates of recombinant *E. coli* by affinity chromatography. Glutathione-sepharose was charged with each GST-HasA, GST-IsdX1 or GST and then incubated with hemoglobin. Resin was sedimented by centrifugation, separated from supernatant containing hemoglobin, washed and bound proteins eluted (Fig. 5). Eluate was analyzed for heme binding by measuring the absorption spectrum of GST-HasA, GST-IsdX1 and GST for heme. GST-HasA and GST-IsdX1 displayed a similar ability to remove heme from hemoglobin. Thus, it seems plausible that IsdX1 functions as a hemophore for *B. anthracis* heme scavenging.

### IsdX1 binds hemoglobin, but not apo-hemoglobin

**Figure 6 ppat-1000132-g006:**
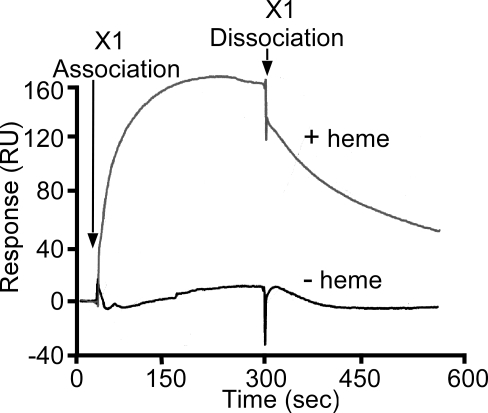
Association and dissociation of IsdX1 and hemoglobin. Interactions between IsdX1 with hemoglobin (with bound heme) or apo-hemoglobin (lacking heme) were assessed by surface plasmon resonance (SPR).

When analyzed by spectrophotometry for absorption at 404 nm, IsdX1 bound heme with an affinity significantly lower than the affinity of apo-hemoglobin for heme (*K_a_*>10^11^ M^−1^) [35]. We therefore considered the possibility that IsdX1 may retrieve heme from hemoglobin by a mechanism that involves physical contact between both proteins [36]. Surface plasmon resonance (SPR) spectroscopy was used to measure the presumed physical association between IsdX1 and hemoglobin [37],[38]. Infusion of IsdX1 over hemoglobin coated chips produced a large spike in the local light refraction index (RU), indicative of a physical interaction between IsdX1 and hemoglobin. This association was saturated within ∼180 seconds and, when deprived of further IsdX1 infusion (arrow), decayed to near baseline RU values (Fig. 6, + heme). Infusion of IsdX1 over chips coated with apo-hemoglobin failed to reveal a physical association between both proteins (Fig. 6, − heme). Following removal of heme from hemoglobin by IsdX1, additional infusion of heme over apo-hemoglobin produced holo-hemoglobin (data not shown), suggesting the inability of IsdX1 to associate with apo-hemoglobin is not caused by the unfolding of this polypeptide. Physical interaction between IsdX1 and hemoglobin occurred in a dose-dependent manner that could be saturated as the concentration of IsdX1 increased (Fig. S3). Dissociation constants for the interaction between IsdX1 and hemoglobin are 7.33×10^−6^ M (holo-hemoglobin) and 9.43×10^−3^ M (apo-hemoglobin). Thus, IsdX1 appears to bind directly to hemoglobin and, upon transfer of heme, dissociates from apo-hemoglobin.

### Hemophore function and specificity in the Isd pathway

**Figure 7 ppat-1000132-g007:**
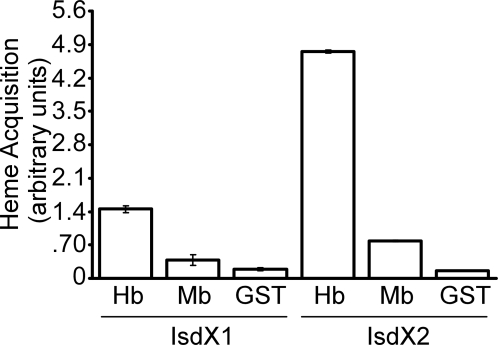
Specificity of IsdX1 and IsdX2. Heme acquisition when equimolar amounts of hemoglobin (Hb) or myoglobin (Mb) [800 µM] were incubated with GST-IsdX1 or GST-IsdX2 (60 µM). Mean and standard deviation of three independent experiments are recorded.

To examine the specificity of IsdX1 and IsdX2 for host hemoproteins, GST-IsdX1/-IsdX2 were incubated with excess hemoglobin and myoglobin, a monomeric globin abundantly present in muscle tissue [39]. As compared to hemoglobin, GST-IsdX1/-IsdX2 displayed little hemophore activity towards human myoglobin (Fig. 7), and similar results were observed when bovine or equine myoglobin was examined (data not shown). These data suggest that during *B. anthracis* infection IsdX1 and IsdX2 most likely prefer hemoglobin over myoglobin as a heme source.

**Figure 8:**
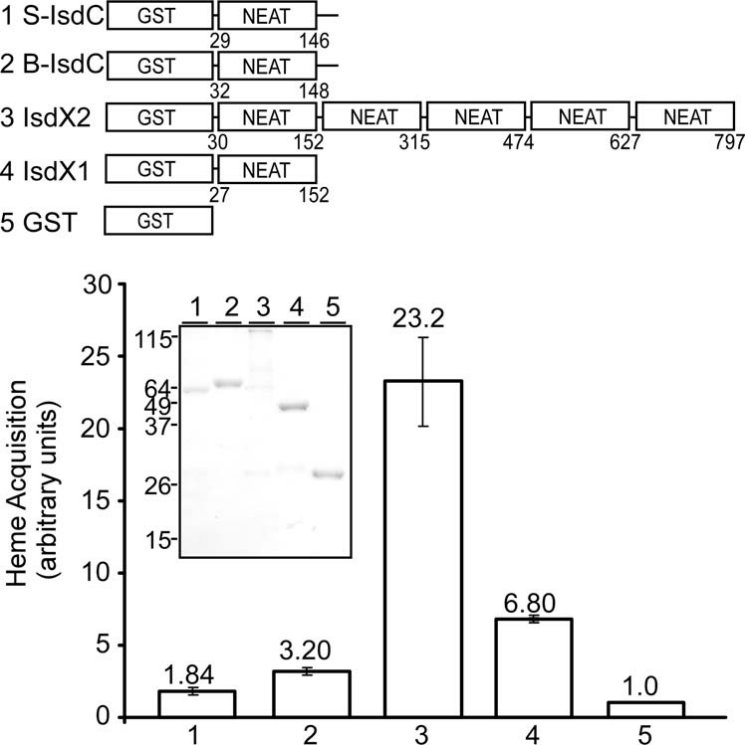
Transfer of heme from hemoglobin to NEAT domain proteins. Purified GST hybrids with (1) *S. aureus* IsdC (S-IsdC), (2) *B. anthracis* IsdC (B-IsdC), (3) IsdX2, (4) IsdX1, or (5) GST control were incubated with hemoglobin and heme transfer measured as in Fig. 4. Mean and standard deviation of three independent experiments are recorded. Inset reveals the mobility of purified proteins on Coomassie stained SDS-PAGE.

Almost the entire IsdX1 polypeptide is comprised of its NEAT domain (Fig. 3A). To test whether other NEAT domain proteins also display hemophore activity, GST fusions to *S. aureus* IsdC and *B. anthracis* IsdC were purified and compared to GST-IsdX1/-X2 (Fig. 8). All four hybrids were able to remove heme from hemoglobin. IsdX2, which contains 5 NEAT domains, was 3.4 fold more efficient than IsdX1 and 7.25 or 12.6 fold more effective than *B. anthracis* IsdC or *S. aureus* IsdC. Also, hemoglobin was not sedimented in any of the reactions, suggesting a transient association similar to that observed for IsdX1 (Fig. 8, inset). Thus, the direct acquisition of heme from hemoglobin appears to be a general property of some NEAT domain proteins, albeit that IsdX1 and IsdX2, when compared to IsdC, clearly display superior activity. This finding is compatible with their localization to the extracellular milieu, a site expected to optimize their interaction with hemoglobin.

### *B. anthracis* IsdX1 scavenges heme from hemoglobin in vivo

**Figure 9:**
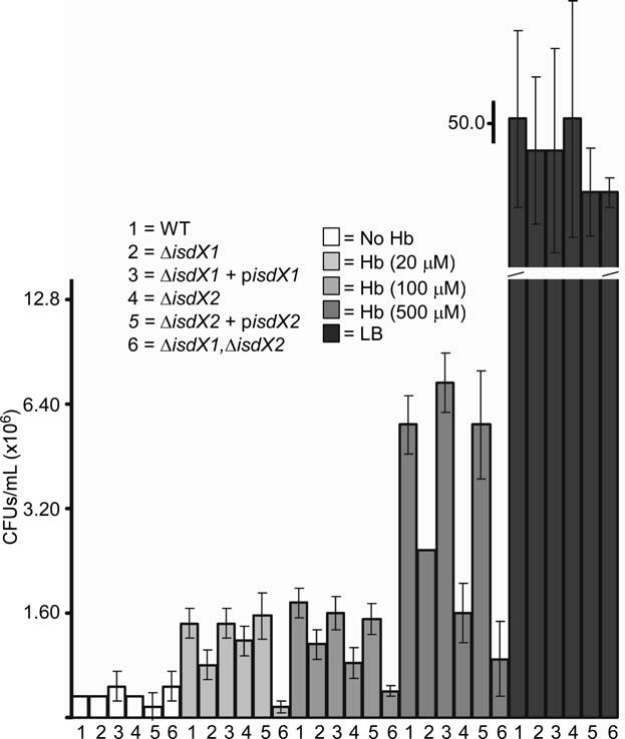
*B. anthracis* requires *isdX1* and *isdX2* for scavenging of heme from hemoglobin. *B. anthracis* strain Sterne (wild-type – WT) and isogenic Δ*isdX1*, Δ*isdX2*, or Δ*isdX1/isdX2* variants were transformed with pLM5 vector, p*isdX1* or p*isdX2* (encoding IsdX1 or IsdX2 proteins, respectively). Bacteria were grown in iron-replete (LB) or iron-deplete medium (IDM) with the indicated concentrations of hemoglobin at 30°C for 16 hours. *B. anthracis* growth was assayed by plating 5 µL of a 1∶400 dilution of bacterial culture onto LB/ Km agar plates and colony forming units per mL (CFUs/mL) determined. Mean and standard deviation of three independent experiments are recorded.

To examine whether the *in vitro* biochemical activity ascribed to IsdX1 and IsdX2 correlated with *in vivo* biological function, wild-type, Δ*isdX1*, Δ*isdX2*, and Δ*isdX1*/Δ*isdX2* mutant *B. anthracis* strains were analyzed for growth in iron defined media (IDM) with hemoglobin as the only source of iron [40]. In the absence of added hemoglobin, all strains grew very poorly in IDM (Fig. 9). The addition of increasing amounts of hemoglobin allowed wild-type *B. anthracis* to grow with increasing rates (Fig. 9), indicating that bacilli can utilize hemoglobin as a source of iron. All three mutant strains (Δ*isdX1*, Δ*isdX2*, and Δ*isdX1*/Δ*isdX2*) displayed a growth defect under iron-depleted conditions with hemoglobin as the sole iron source (Fig. 9). Whereas deletion of individual genes, *isdX1* or *isdX2*, caused a reduction in growth, these defects were exacerbated for the double mutant strain, which is unable to secrete IsdX1 or IsdX2 (Fig. 9 and Fig. S4). These data suggest *isdX1* and *isdX2* perform partially overlapping functions in the heme scavenging pathway of bacilli. Growth defects of Δ*isdX1* and Δ*isdX2* mutants were restored when bacilli were transformed with plasmids providing for IPTG inducible expression of each respective gene. Finally, all strains examined grew equally well in iron-replete media (Fig. 9, far right columns). Collectively, these experiments suggest that *B. anthracis* IsdX1 and IsdX2 function as secreted hemophores for heme-scavenging from hemoglobin.

## Discussion

The ability of mammalian organisms to sequester iron and limit its availability serves as a defense against microbial infection [41]. Iron is stored intracellularly, where ferric iron is complexed by ferritin or incorporated by ferrochelatase into porphyrin. The resulting product, heme, is bound by hemoproteins, e.g. hemoglobin or myoglobin [42]. Dedicated traffic systems for ferric iron (transferrin) or heme (hemopexin) transport iron in body fluids between tissues. A key feature that enables bacteria to replicate within their hosts is the production of siderophores, iron-sequestering compounds that scavenge iron from transferrin, and synthesis of cognate siderophore transport systems for the bacterial envelope [43]. Vertebrates, in turn, evolved defense mechanisms that exploit the bacterial requirement for iron by producing lipocalin, siderocalin or related proteins which sequester iron [44].

*B. anthracis* employs two siderophores to retrieve ferric-iron during infection, bacillibactin and petrobactin (anthrachelin) [45],[46]. Petrobactin, enzymatically derived from 3,4-dihydroxybenzoate, spermidine and citrate via products of the *asbA-F* locus, is essential for *B. anthracis* growth, as mutations in *asbA-F* cause significant defects in the pathogenesis of anthrax [40],[47],[48]. Interestingly, this siderophore is resistant to sequestration by siderocalin, an immune protein which binds siderophores as a bacterial defense strategy [49]. *B. anthracis* has also evolved a scavenging pathway for heme that is encoded by the *isd* locus (*isdC-isdX1-isdX2-isdE-isdE2-isdF-srtB-isdG*) [25]. IsdC, a NEAT domain protein with C-terminal sorting signal, is anchored to cell wall peptidoglycan by sortase B (SrtB) [25]. IsdE-IsdE2-IsdF membrane transporter is thought to import heme into bacterial cells, while IsdG, a cytoplasmic monooxygenase, cleaves the tetrapyrrol of heme, thereby liberating iron [24].

Heme scavenging strategies of *B. anthracis* must take into account the unique envelope attributes of this pathogen. Bacilli evolved a thick murein sacculus comprised of peptidoglycan with attached envelope polymers: poly-D-glutamic acid (PDGA) capsule, carbohydrate polysaccharide, teichoic acid and proteins [12]. Further, bacilli elaborate S-layers, two-dimensional crystalline arrays of proteins bearing SLH domains that are immobilized by interaction with pyruvylated cell wall polysaccharide [11],[50]. It is not certain that bacilli elaborate all envelope components at each stage of infection [51]. Nevertheless, explosive growth of *B. anthracis* and the accompanying need for nutrients likely demand that heme scavenging pathways must engage all structural components of the bacterial envelope. Here we report that *B. anthracis* secretes two polypeptides, IsdX1 and IsdX2, into the extracellular milieu. The absence of a canonical sortase recognition motif in the C terminus of IsdX2 suggests it is not anchored to the cell wall by a sortase. Both proteins remove heme from hemoglobin, thereby enabling *B. anthracis* growth under conditions when hemoglobin is the sole source of iron. These findings, along with the data presented in Figures 4–8, suggest one of the functions of the NEAT domain is the direct acquisition of heme from hemoglobin. How IsdX1 and IsdX2 bind heme is currently unknown; however, studies from other NEAT proteins suggest that heme-iron is ligated by a conserved tyrosine with high spin, five-coordinate geometry [20],[52],[53].

It seems unlikely that IsdX1 or IsdX2 deliver heme directly to the bacterial membrane, as the cell wall envelope cannot be penetrated by proteins. Instead, IsdX1 and IsdX2 probably transfer heme to other NEAT domain proteins at strategic positions throughout the bacterial envelope, a hypothesis consistent with their secretion into the surrounding milieu. In agreement with this conjecture, *in silico* analysis of the *B. anthracis* genome identified several genes encoding NEAT domain proteins with variable envelope locations: peptidoglycan linked IsdC [25],[54], BasJ positioned in the plasma membrane [55], and BslK, an S-layer protein [56]. In contrast to the complex features of the envelope in bacilli, staphylococci, listeria and clostridia are much simpler and cannot elaborate a large capsule or S-layer [18]. Not surprisingly, these microbes are capable of scavenging heme with NEAT domain proteins that are exclusively immobilized in cell wall peptidoglycan.

Heme scavenging pathways in Gram-negative bacteria have been studied in great detail. *S. marcescens* employs a type I secretion machine (HasDEF) and recognition of a C-terminal secretion signal to transport HasA across the bacterial double membrane envelope [32],[57],[58]. By virtue of its unique structure and affinity for ligand (*K_a_* 5×10^10^ M^−1^), HasA retrieves heme from hemoglobin, myoglobin or hemopexin [30],[59],[60],[61] and delivers the compound to HasR, the outer membrane receptor. Although HasR has much lower affinity for heme (*K_a_* 5×10^6^ M^−1^), the outer membrane receptor receives heme from HasA by a mechanism involving physical interactions between both proteins [60],[61]. TonB(HasB)-ExbB-ExbD dependent relay then transfers heme from HasR across the periplasm, initiating subsequent import into the cytoplasm [62]. HasA production and secretion are regulated by an ECF type sigma factor (HasI) and its cognate anti-sigma factor (HasS) [63]. Biological activities of HasI/HasS are informed by reciprocal associations between HasA, HasR and heme [64]. Hemophore systems with similar design exist in *Haemophilus influenzae* [65], *Yersinia enterocolitica* [66], and *Pseudomonas aeruginosa* [67],[68],[69]. Pathogenic *Neisseria spp.*, on the other hand, elaborate outer membrane proteins that not only bind hemoproteins but also remove heme. IsdX1 represents the first secreted hemophore in Gram-positive bacteria, a finding that invites a functional comparison with HasA, the secreted hemophore of Gram-negative microbes [31]. Unlike HasA, which acquires heme from diverse hemoproteins such as myoglobin, IsdX1 appears to be specific for hemoglobin [30]. Further, whereas HasA seems to acquire heme from hemoglobin by virtue of its higher affinity for heme [31],[60], IsdX1 directly associates with hemoglobin for extraction of the heme. Finally, the structure of HasA is quite distinct from that of other NEAT-domain proteins [20],[53],[70],[71]. These findings suggest that the molecular mechanism whereby IsdX1 acquires heme from hemoglobin must be distinct from that of HasA. While HasA delivers heme to outer-membrane receptors [60],[61], secreted components of the *isd* locus encoding NEAT domain proteins, such as IsdX1, provide a versatile strategy for stealing heme that can be adapted to unique microbial envelope structures of Gram-positive pathogens. Whether these specific adaptations are important during infections caused by Gram-positive pathogens, e.g. *B. anthracis*, is a topic currently being explored in our laboratory.

## Materials and Methods

### Bacterial strains and reagents

*B. anthracis* strain Sterne 34F2 [72] and *E. coli* strains (DH5α, XL1-Blue or K1077) were grown in Luria-broth (LB) or brain-heart infusion (BHI) (Table S1). Antibiotics were used for plasmid selection (ampicillin 50 µg/ml, kanamycin 20 µg/ml). All reagents were purchased from Sigma unless otherwise noted. *B. anthracis* chromosomal DNA was extracted using the Wizard Genomic DNA Purification Kit (Promega). The *isdX1* gene (BAS4443) of *B. anthracis* Sterne was deleted by allelic replacement with the temperature- sensitive pLM4 [26]. Briefly, 1,000 bp of 5′ and 3′ *isdX1*-flanking sequences were PCR amplified with primer pairs *isdX1*-EcoRI (5′-gatcgatcgaattgattttcattgagaatgataatc-3′) and *isdX1*-SacI (5′-gatcgatcgagctcttgtttaaacatatattcatcacc-3′) as well as *isdX1*-SacI (5′-gatcgatcgagctcgggaacagtattaaataattttc-3′) and *isdX1*-KpnI (5′-gatcgatcggtacccctctggttgtttctcttc-3′). Following ligation, the 2-kb inset was cloned between the EcoRI/KpnI sites of pLM4 to create pLM4-Δ*isdX1*. After transformation into BAS7, bacilli were grown first at 30°C (permissive temperature) on LB/Km and then shifted to 43°C (restrictive temperature), followed by growth at 30°C to induce plasmid loss, thereby generating BAS8. DNA was analyzed for the presence of *isdX1* by PCR and deletions confirmed by DNA sequencing. Deletion of *isdX2* (BAS4442) was achieved as previously reported [25]. The deletion of both *isdX1* and *isdX2* in the same strain was achieved via the procedure described above for Δ*isdX1* using the Δ*isdX1* 5′ flank primers and the following 3′ flank primers: *isdX2*-SacI (5′-gatc gatcgagctcctagttcgtaaatatagagcagg-3′) and *isdX2*-KpnI (5′-gatcgatcggtaccccttgtacaagttc aacaatacc-3′). Plasmid DNA was amplified in *dam* mutant *E. coli* strain K1077 prior to electroporation of bacilli [73].

### Protein purification

Signal peptides of IsdX1, IsdX2, B-IsdC, and Sa-IsdC were replaced with glutathione S-transferase (GST) and recombinant proteins were purified by GST-affinity chromatography (see *Protocol S1*).

### IsdX1 secretion

Overnight *B. anthracis* cultures were incoculated into 2 ml of BHI (+ Fe) or chelex-treated BHI (− Fe) supplemented with Ca^2+^, Mg^2+^, Mn^2+^, and Zn^2+^ and incubated at 37°C for further growth [25]. Bacilli were sedimented by centrifugation at 10,000×*g*, washed twice with 1 ml of PBS (pH 7.4) and fractionated as previously reported [25]. Samples were analyzed by immunoblot with αL6, αSrtB, αIsdC, or αIsdX1 specific rabbit antisera (1∶1,000), followed by mouse anti-rabbit HRP-linked antibody (1∶10,000) and ECL (enhanced chemiluminescence, Pierce, Rockford, IL). By comparing the amount of secreted IsdX1 and IsdX2 to a known amount of recombinant purified IsdX1/X2 via immunoblot, we estimate that a 3 mL culture of *B. anthracis* containing an optical density of 1.0 will secrete 0.52±0.25 µg of total IsdX1 in 12 hours. This compares to 0.55±0.07 µg of total IsdX2 secreted under the same conditions.

### Heme binding to IsdX1 and IsdX2

IsdX1 (20 µM) or IsdX2 (1 µM) were incubated in 50 mM Tris-HCl, pH 8.0 with or without hemin chloride (0.01–40.0 µM in 0.1 M NH_4_OH) for 5 minutes at 25°C, followed by spectrophotometry (300–700 nm) in a Varian Cary 50BIO instrument. Peak absorbance at 404 nm, characteristic of heme binding, was monitored following subtraction of a hemin-only reference cuvette value at each concentration.

### IsdX1 acquisition of heme from hemoglobin

GST-IsdX1 (60 µM) or PBS (control) was incubated with 50 µL of glutathione-sepharose (Amersham) for 30 min at 25°C, followed by 3 washes of 200 µL with PBS. Bovine hemoglobin (Sigma H2500) was added to 60 µM (monomer) and the X1/Hb mixture was incubated for 30 min at 25°C. Reactions were centrifuged at 13,000×*g* to sediment glutathione-sepharose/GST-IsdX1 complexes, reactions washed three times with 200 µL of PBS and GST-IsdX1 eluted in 50 µL of 600 mM reduced glutathione (pH 8.0). Sediment (GST-IsdX1) and supernatant (hemoglobin) were analyzed by spectroscopy and heme binding quantified by measuring absorbance at 404 nm. For [^55^Fe]heme transfer, reactions were prepared as indicated above except that the amount of GST-IsdX1 added varied from 0.1–140 µM (see *Protocol S1*). The amount of [^55^Fe]heme in the sediment (GST-IsdX1/resin) or supernatant (hemoglobin) was quantified in a Beckman LS-6000IC instrument (Beckman-Coulter, Fullerton, CA). Percent amount of heme was calculated by dividing the counts in the sediment or supernatant by the total number of counts in each reaction multiplied by 100. For the experiment presented in Fig. 7, the heme-transfer assay was utilized with the concentrations of hemoglobin and myoglobin (Sigma M0630) at 800 µM. Heme acquisition was calculated as follows: [(GST-IsdX1_Abs.404nm_) minus (glutathione-sepharose(background)_Abs.404nm_) divided by [total input_Abs.404nm_] times 100.

### SPR analysis

IsdX1-hemoglobin interactions were measured with a BIAcore 3000 biosensor (GE Healthcare) via surface plasmon resonance (SPR) [37],[38]. Hemoglobin, 180 pmol in HBS (10 mM HEPES, pH 7.4, 0.15 M NaCl, 50 mM EDTA, 0.05% Tween 20), was amine coupled to CM5 sensor chip at 25°C at a flow rate of 5 µL/min [74]. Hemoglobin injection was stopped once response was saturated at 2,100 RU and 50 µM IsdX1 in HBS was infused at 20 µL/min with a dissociation time of 300 sec at 25°C. Data were fit to a model of equimolar IsdX1-hemoglobin association with BIAevaluation version 4.1. A dose-dependent response was observed over an IsdX1 concentration range of 3–50 µM.

### *B. anthracis* growth with hemoglobin

Bacilli from overnight cultures in 2 ml of LB+Km at 30°C were inoculated into IDM+Km [40], grown for 12 hours at 30°C, bacteria harvested by sedimentation at 10,000×*g*, washed twice and then suspended in 1 ml IDM (O.D. 4.0). Aliquots (5 µL) were inoculated into 150 µL IDM, with or without Hb (20, 100, or 500 µM) using 96-well U-bottom plates (Corning, Corning, NY). After 16 hours of incubation at 30°C, growth was assayed by plating 5 µL of a 1∶400 dilution of bacterial culture onto LB/ Km agar plates and colony forming units per mL (CFUs/mL) determined. For plasmid complementation, 1.5 mM IPTG (final concentration) was added to culture media. A list of accession numbers (NCBI) for genes in this study are as follows: *isdX1* = YP_030690, *isdX2* = YP_030689, *b-isdC* = YP_030691, *isdC* = YP_001332076.

## Supporting Information

Figure S1 Expression of IsdX1 and IsdX2 at different temperatures
(0.19 MB DOC)

Figure S2 Heme binding to IsdX1 at different temperatures
(0.09 MB DOC)

Figure S3 Association of IsdX1 and hemoglobin
(0.13 MB TIF)

Figure S4 Expression of IsdX1, IsdX2, and B-IsdC in Δ*isdX1/isdX2 B. anthracis*
(0.08 MB DOC)

Protocol S1 Supplementary Materials and Methods, References, and Figure Legends
(0.05 MB DOC)

Table S1 Bacterial strains used in this study
(0.04 MB DOC)
