# Supplementary figures and images for: Bacillus anthracis Secretes Proteins That Mediate Heme Acquisition from Hemoglobin
Source: PLoS Pathog. 2008 Aug 22;4(8):e1000132. doi: 10.1371/journal.ppat.1000132 (PMC2515342; doi:10.1371/journal.ppat.1000132)

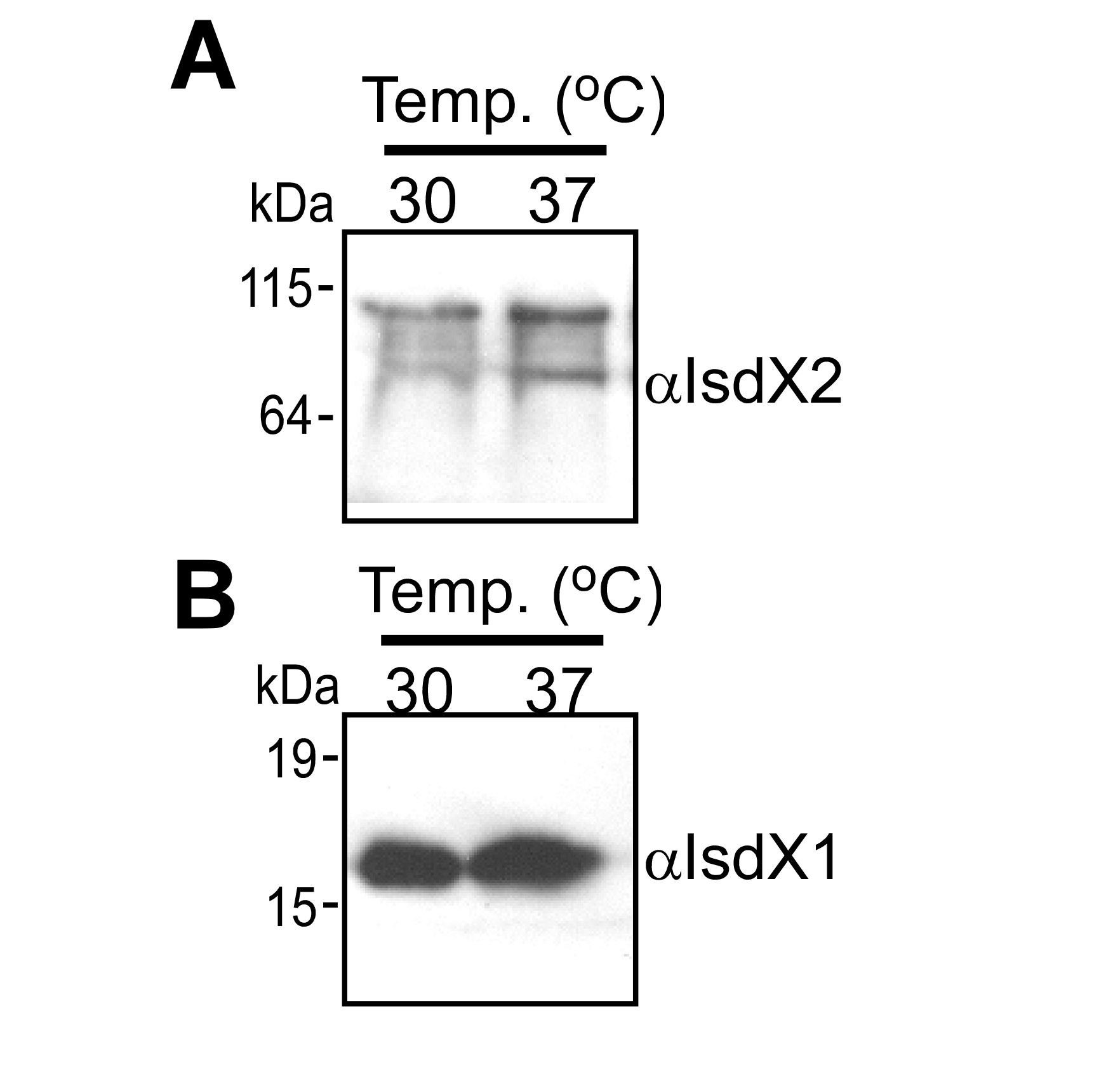

Supplement: Figure S1 — Expression of IsdX1 and IsdX2 at different temperatures (0.19 MB DOC) [file ppat.1000132.s001.tif]

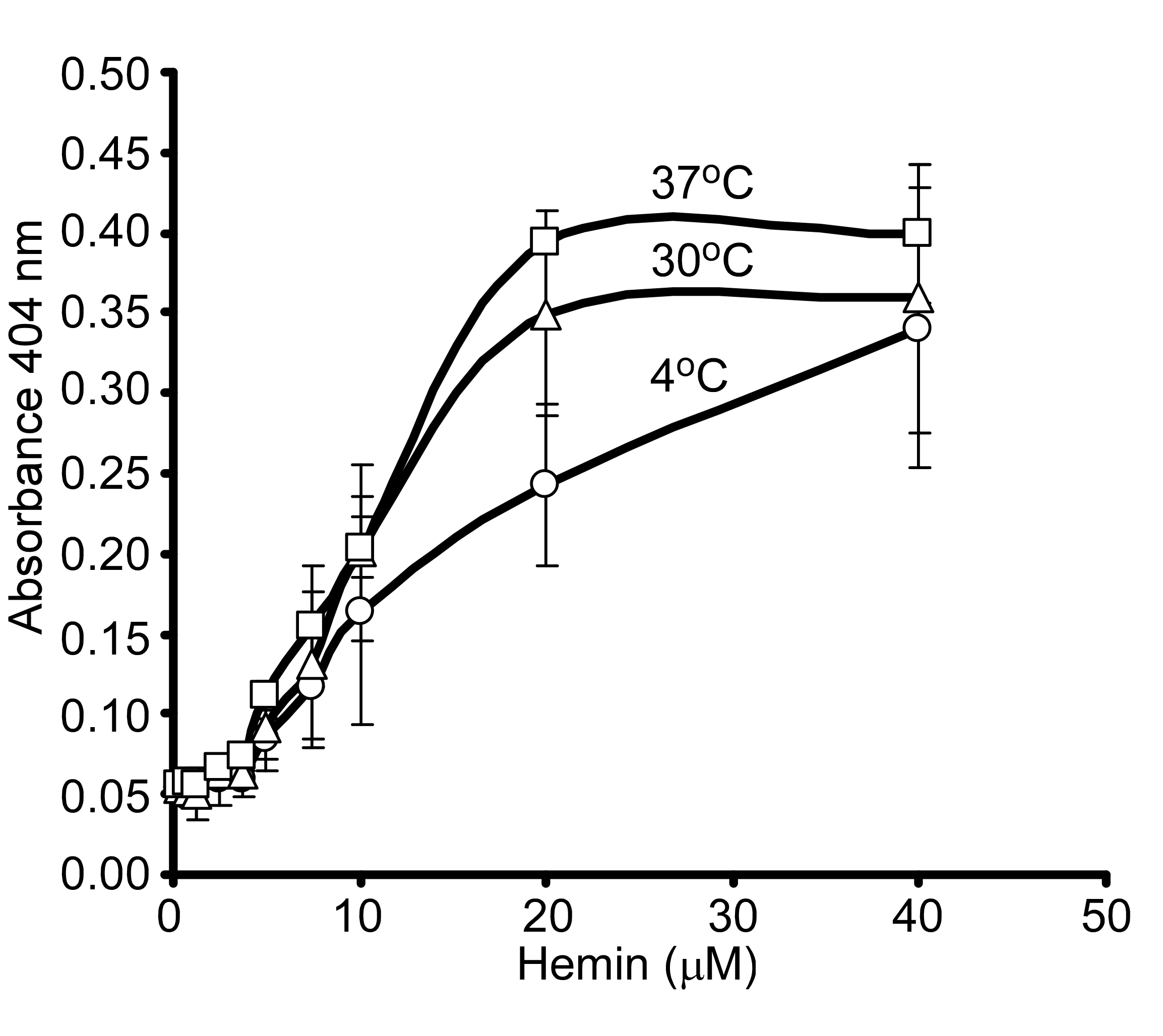

Supplement: Figure S2 — Heme binding to IsdX1 at different temperatures (0.09 MB DOC) [file ppat.1000132.s002.tif]

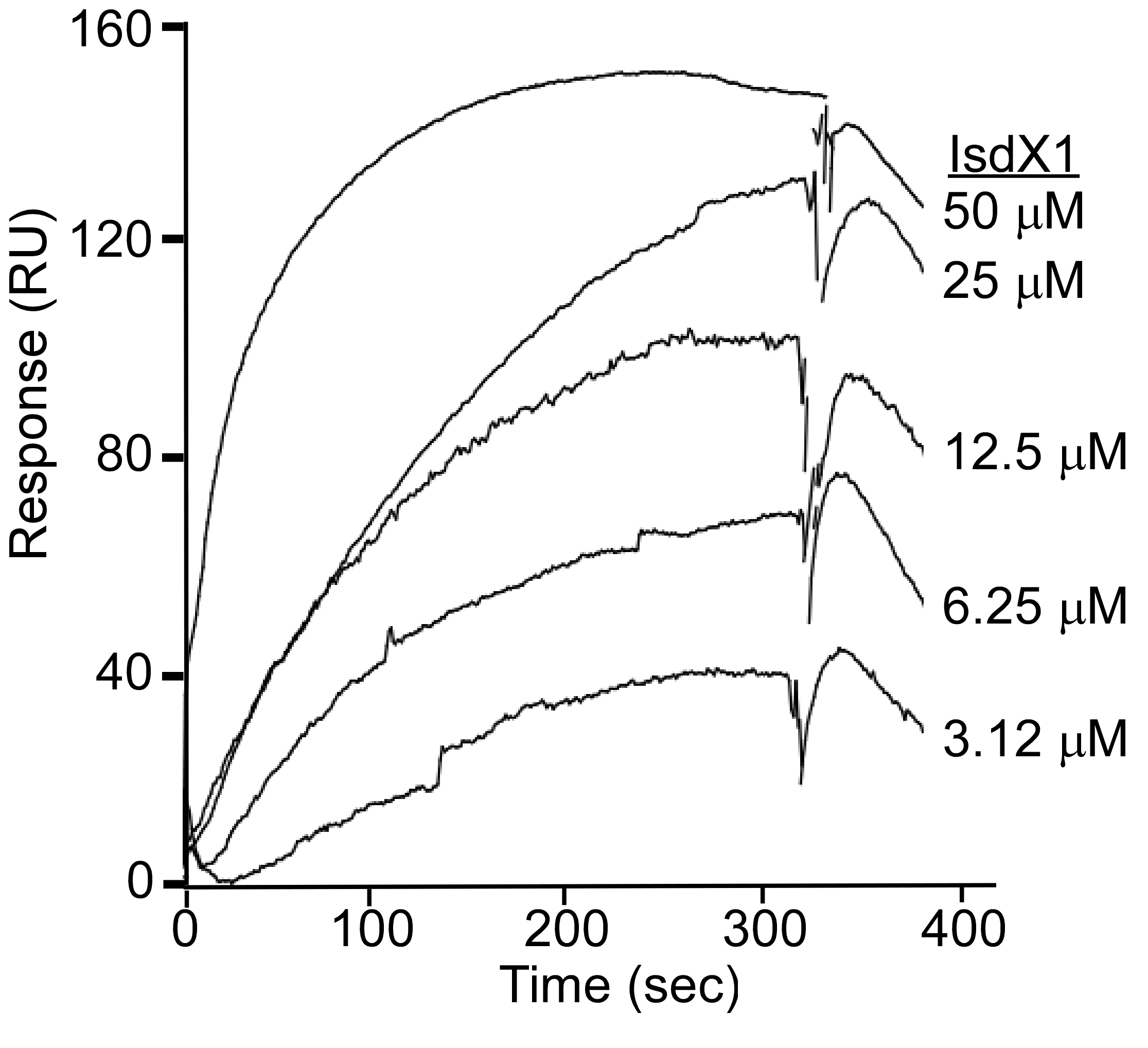

Supplement: Figure S3 — Association of IsdX1 and hemoglobin (0.13 MB TIF) [file ppat.1000132.s003.tif]

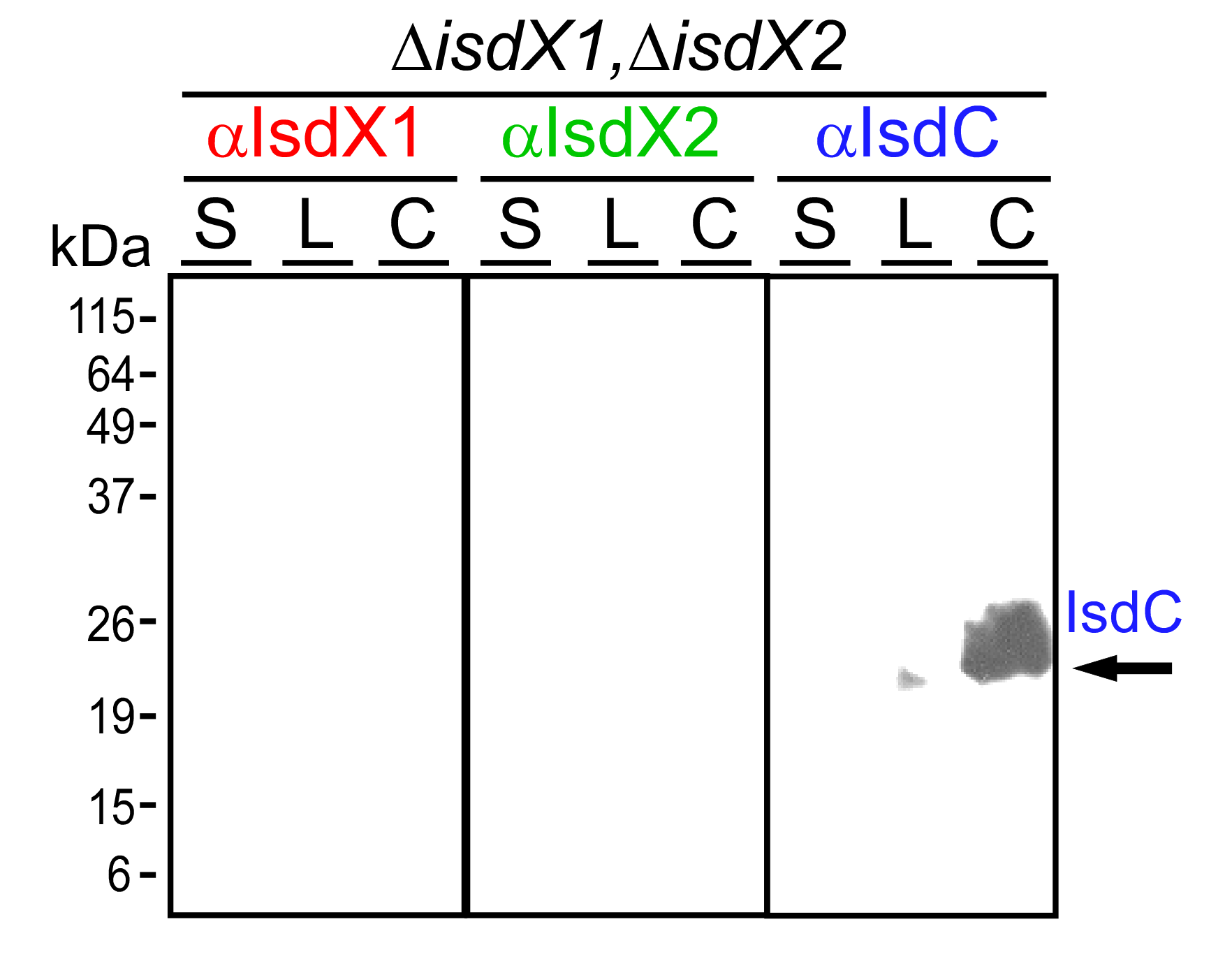

Supplement: Figure S4 — Expression of IsdX1, IsdX2, and B-IsdC in ΔisdX1/isdX2 B. anthracis (0.08 MB DOC) [file ppat.1000132.s004.tif]
