# Supplementary material for: Bacillus anthracis Secretes Proteins That Mediate Heme Acquisition from Hemoglobin
Source: PLoS Pathog. 2008 Aug 22;4(8):e1000132. doi: 10.1371/journal.ppat.1000132 (PMC2515342; doi:10.1371/journal.ppat.1000132)
Supplement: Protocol S1 — Supplementary Materials and Methods, References, and Figure Legends (0.05 MB DOC) [file ppat.1000132.s005.doc]

***Supplementary Material***

***Bacillus anthracis* secretes proteins that mediate heme acquisition from hemoglobin**

Anthony W. Maresso, Gabriella Garufi, and Olaf Schneewind**^*^**

Department of Microbiology, University of Chicago, Chicago, IL, USA

**^*^**Corresponding author. Department of Microbiology, University of Chicago, 920 E. 58^th^ Street, Chicago, IL 60637, USA Tel.: +1 773 834 9060; Fax: +1 773 834 8150; E-mail: [oschnee@bsd.uchicago.edu](mailto:oschnee@bsd.uchicago.edu)

**Supplementary** **Materials and Methods**

*Protein purification*

Expression plasmid p*gst-isdX1* was constructed by PCR amplification of *isdX1* (amino acids 27-152) sequence from the *B. anthracis* genome using the primer pair *gst-isdX1*-BamHI-1-(5'-gatcgatcggatccgcaaaggctgctacaaaactag-3') and *gst-isdX1*-EcoRI-1-(5'-gatcgatcgaattcttatttaatactgttcccatc-3'). Expression plasmid p*gst-isdX2* was constructed by PCR amplification of *isdX2* sequence (amino acids 30-859) from the *B. anthracis* genome using the primer pair *gst-isdX2*-EcoRI-1-(5'-gatcgatcgaattcacgct

accaagttggctgacgg -3') and *gst-isdX2*-EcoRI-1-(5'- gatcgatcgaattcaattgctgtatctgctgtttttg -3'). Expression plasmid p*gst-HasA* was constructed by PCR amplification of *HasA* sequence (amino acids 1-170) from pBGS18 (a gift from C. Wandersman) using the primer pair *gst-HasA*-BamHI-1-(5'-gtacgtacggatccgcattttcagtcaattatgacag -3') and *gst-HasA*-EcoRI-1-(5'- gatcgatcgaattcaatcgccgccacctgatcgaag -3'). Product DNA was digested with BamHI or EcoRI and cloned into pGEX-2TK (Amersham). *E. coli* XL-1 Blue harboring p*gst-isdX1* (EC1), p*gst*-*isdX2* (EC2) or p*gst*-*hasA* (EC3) were propagated on LB agar with ampicillin overnight at 37°C. Bacteria scraped from agar plates were inoculated into 1 L fresh LB and rotated with 250 rpm at 37°C. After 2 hours of incubation, 1.5 mM isopropyl-ß-D-thiogalactopyranoside (IPTG) was added and cultures were incubated for an additional 2 hours. Bacteria were sedimented by centrifugation at 6,000 ×*g* for 8 min, suspended in binding buffer (50 mM Tris-HCl, pH 7.9, and 150 mM NaCl), and cells broken with two passes through a French press at 14,000 psi. Bacterial lysates were centrifuged at 30,000 ×*g* for 20 min, and soluble protein in supernatants was passed through a 0.45-µm pore-size cellulose filter. Filtrates were subjected to affinity chromatography on 2-ml GST-resin (Amersham), column pre-equilibrated with 20 ml of binding buffer. Columns were washed with 40 ml binding buffer and target proteins eluted with 2 ml binding buffer with 25 mM reduced glutathione. Purified proteins were dialyzed against at least 3 L of PBS (pH 7.4) for 3-4 days and stored at -20°C. It is estimated <10% of the total purified IsdX1 or IsdX2 is bound to iron-porphyrin from *E. coli*. Most of the residual heme was removed by dialysis. When necessary, the GST-tag was removed from IsdX1 and IsdX2 by thrombin digestion according to the manufacturer’s instructions (Amersham). B-IsdC and S-IsdC were purified as previously described [1,2].

*Secretion of recombinant IsdX1*

Expression plasmids p*isdX1_H6_*, p*isdX1*, and p*isdX2* were constructed by PCR amplification of *isdX1* and *isdX2* from the *B. anthracis* genome using the primer pairs: *isdX1_H6_*-KpnI-forward (5'-gatcgatcggtaccgggtgatgaatatatgtttaaac-3')/ *isdX1_H6_*-KpnI-reverse (5'-ctagctagggtaccttagtgatggtgatggtgatgtttaatactgttcccatcaaatac-3') and the *isdX1_H6_*-KpnI-forward with *isdX1-*KpnI-reverse (5'-catgcatgggtaccttatttaata

ctgttcccatcaaatac-3'), 5' segment-*isdX2*-XbaI-forward (5'-gatcgatctctagattgaaca

ggtatacaaaaatcattg-3')/*isdX2*-EcoRI/KpnI-reverse (5'-catgcatgggtaccctagctaggaatt

ccatcttttacttttagcaaacc-3') and 3' segment-*isdX2*-EcoRI-forward (5'-gatcgatcgaattcgtaa

gaagtatgtatcatttacg-3')/*isdX2*-KpnI-reverse (5'-catgcatgggtaccccaaaaattacaatctacctgc-3'). The DNA was cloned into pLM5. *B. anthracis* strain Sterne 34F2 transformed with p*isdX1_H6_* (BAS10) or pLM5 vector control (BAS12) were inoculated into 2 ml of BHI (+ Fe) and grown at 37°C for 12 hrs. After the addition of IPTG to a final concentration of 1.5 mM, bacilli were grown for 2 additional hours, then harvested by centrifugation and fractionated as previously described [1]. *B. anthracis* Δ*isdX2* strain was transformed with pLM5 (BAS14) or p*isdX2* (BAS15) while the Δ*isdX1*/Δ*isdX2* strain was transformed with pLM5 (BAS16).

*B. anthracis infection of guinea pigs*

To determine if IsdX1 and IsdX2 are expressed during anthrax disease, *B. anthracis* strain Ames infection of Guinea pigs was performed [3]. Briefly, *B. anthracis* spores were prepared by growing the Ames strain in sporulation media for 6 days to induce sporulation. Spores were treated at 65°C for 60 min to kill vegetative cells, and the spore suspension examined for colony formation. A dilution of 10-100 spores in 0.1 mL of PBS were injected subcutaneously into the right hind leg of two male, eight-week old guinea pigs and progression to acute disease monitored over a fourteen day time period. At day five and ten, ciprofloxacin was administered to ensure animal survival. At day fourteen, animals were bled via heart puncture and serum isolated via Sarstedt Microtubes according to the manufacturer’s instructions. Approximately 50 pmoles of GST-X1, GST-X2, or GST were analyzed by immunoblot using freshly isolated sera from a single animal at a 1:2,000 dilution, followed by addition of anti-Guinea pig HRP-conjugated antibody at a 1:5,000 dilution. All sera were stored at -20°C. Animal experiments were performed in accordance with institutional guidelines following experimental protocol review and approval by the Institutional Biosafety Committee and the Institutional Animal Care and Use Committee.

*[^55^Fe]heme- hemoglobin*

[^55^Fe]heme-hemoglobin was prepared by incubating GST-IsdX1 bound to GST resin with hemoglobin (molar ratio of GST-IsdX1:hemoglobin = 5:1) for 1 hour at 25ºC. GST-IsdX1 was sedimented by centrifugation at 13, 000 ×*g* and hemoglobin assayed for the loss of heme by Soret spectroscopy. Under these conditions, most of the heme is removed from Hb. Apo-Hb was immediately incubated with 5 μL (~ 100 μCi) [^55^Fe]heme (RI Consultants, Hudson, NH) for 1 hour at 25ºC, followed by dialysis against 1 L of PBS, pH 7.4 at 4ºC for 24 hours to remove unbound [^55^Fe]heme. [^55^Fe]heme-hemoglobin was stored at -20 ºC.

*Hemophore activity of NEAT domain proteins*

NEAT domain proteins GST-IsdC (S), - IsdC (B), -IsdX2, -IsdX1, or GST alone (200 μM) were incubated with excess Hb (800 μM) as described for the heme transfer assay. Heme acquisition was calculated by multiplying the initial heme measurement prior to the reaction (GST_Abs.404nm_/NEAT-protein_Abs.404nm_) by the heme measurement after the reaction (NEAT-protein_Abs.404nm_/GST_Abs.404nm_) normalized to GST (assigned a value of 1). For comparison of IsdX1 to HasA, experiments were performed as described for the heme-transfer assay except the Hb concentration was 50, 200, or 800 μM.

**Supplementary References**

1. Maresso AW, Chapa TJ, Schneewind O (2006) Surface protein IsdC and sortase B are required for heme-iron scavenging of *Bacillus anthracis*. J Bacteriol 188: 8145-8152.

2. Mazmanian SK, Skaar EP, Gasper AH, Humayun M, Gornicki P, et al. (2003) Passage of heme-iron across the envelope of *Staphylococcus aureus*. Science 299: 906-909.

3. Marraffini LA, Schneewind O (2006) Targeting proteins to the cell wall of sporulating *Bacillus anthracis*. Mol Microbiol 62: 1402-1417.

4. Sterne M (1937) Avirulent anthrax vaccine. Onderstepoort J Vet Sci Animal Ind 21: 41-43.

**Supplementary Figure Legends**

**Figure S1**. Expression of IsdX1 and IsdX2 at different temperatures. Wild-type *B. anthracis* was grown in LB for 12 hours at 37°C, followed by inoculation into IDM and grown for 16 hours at either 30°C or 37°C. Supernatants from sedimented cultures were analyzed for expression of IsdX1 or IsdX2 by immunoblot analysis (as described in the Methods). The immunoblot is representative of one of three independent determinations.

**Figure S2**. Heme binding to IsdX1 at different temperatures. Heme binding to IsdX1 was performed as described in the legend to Figure 3 with inclusion of a 5 minute incubation step at 4°C, 30°C, or 37°C. The mean and standard deviation of three independent determinations is shown.

**Figure S3**. Association of IsdX1 and hemoglobin. Purified IsdX1 (3-50 μM) was infused over immobilized holo-hemoglobin (~180 pmol were coupled) and interactions assessed by SPR. The sensogram is representative of one of two independent determinations.

**Figure S4**. Expression of IsdX1, IsdX2, and B-IsdC in Δ*isdX1*/*isdX2* *B. anthracis*. *B. anthracis* strain Sterne harboring a deletion in *isdX1* and *isdX2* was grown in IDM for 16 hours at 30°C, separated into secreted (S), lysate (L), and cell wall (C) fractions, and analyzed for expression of IsdX1, IsdX2, and Ba-IsdC according to the legend to Figure 2A and Methods.
