# Supplementary material for: Bacillus anthracis Secretes Proteins That Mediate Heme Acquisition from Hemoglobin
Source: PLoS Pathog. 2008 Aug 22;4(8):e1000132. doi: 10.1371/journal.ppat.1000132 (PMC2515342; doi:10.1371/journal.ppat.1000132)
Supplement: Table S1 — Bacterial strains used in this study (0.04 MB DOC) [file ppat.1000132.s006.doc]

**Supplementary Table**.

|  | | |
| --- | --- | --- |
| **Strain** | **Property** | **Reference** |
| 34F2 | *B. anthracis* Sterne (pX01^+^, pXO2^-^) | [4] |
| BAS7 | 34F2 transformed with pLM4-5’-3’Δ *isdX1* | This study |
| BAS8 | 34F2 Δ*isdX1* | This study |
| BAS9 | 34F2 Δ*isdX2* | This study |
| BAS10 | 34F2 Δ*isdX1* transformed with pLM5-*isdX1_H6_* | This study |
| BAS11 | 34F2 Δ*isdX1* transformed with pLM5-*isdX1* | This study |
| BAS12 | 34F2 transformed with pLM5 | [1] |
| BAS13 | 34F2 Δ*isdX1* transformed with pLM5 | This study |
| BAS14 | 34F2 Δ*isdX2* transformed with pLM5 | This study |
| BAS15 | 34F2 Δ*isdX2* transformed with pLM5-*isdX2* | This study |
| BAS16 | 34F2 Δ*isdX1*/Δ*isdX2* transformed with pLM5 | This study |
| EC1 | *E. coli* XL-1 Blue transformed with p*gst-isdX1* | This study |
| EC2 | *E. coli* XL-1 Blue transformed with p*gst-isdX2* | This study |
| EC3 | *E. coli* XL-1 Blue transformed with p*gst-hasA* | This study |

**Table S1**. Bacterial strains used in this study.
